# Prostate cancer between prognosis and adequate/proper therapy

**Published:** 2017

**Authors:** T Grozescu, F Popa

**Affiliations:** * “Carol Davila” University of Medicine and Pharmacy, Bucharest, Romania

## Abstract

Knowing the indolent, non-invasive nature of most types of prostate cancer, as well as the simple fact that the disease seems more likely to be associated with age rather than with other factors (50% of men at the age of 50 and 80% at the age of 80 have it [**1**], with or without presenting any symptom), the big challenge of this clinical entity was to determine severity indicators (so far insufficient) to guide the physician towards an adequate attitude in the clinical setting. The risk of over-diagnosing and over-treating many prostate cancer cases (indicated by all the major European and American studies) is real and poses many question marks. The present paper was meant to deliver new research data and to reset the clinical approach in prostate cancer cases.

## Epidemiologic data

Data were collected by the National Cancer Institute, USA, within the Surveillance Epidemiology and End Results (SEER) [**1**].

Currently, the mean age of diagnosing the prostate cancer in men is 67, in terms of percentages as follows:

• 0.0% diagnosed at less than 20 years of age

• 0.0% between ages 20 and 34

• 0.6% between ages 35 and 44

• 9.1% between ages 45 and 54

• 30.7% between ages 55 and 64

• 35.3% between ages 65 and 74

• 19.9% between ages 75 and 84

• 4.4% at ages over 85.

The incidence correlated with age was of 156.0 per 100,000 men per year. These data were based on the diagnosed cases from 17 geographical areas in USA monitored by SEER.

**Table 1:** Incidence of men cancer patients in USA

Race/ Ethnicity	Men
All races (globally)	156.0 per 100,000
Caucasians	149.5 per 100,000
Afro-americans	233.8 per 100,000
Asians and Pacific insulars	88.3 per 100,000
Native Americans and from Alaska	75.3 per 100,000
Hispanic	107.4 per 100,000

## Data collected by Cancer Research, UK [**2**]

The incidence of prostate cancer increases steeply with age, with its highest interval between ages 75-79. For men aged 55-59, the incidence was 155 per 100,000. Ten years later, ages 65-69, the incidence was triple, meaning 510 per 100,000. In men aged 75-79, the incidence was five times higher, of 751 per 100,000.

Prevalence: estimates from post-mortem data (no matter the cause of death) showed that half of the men aged 50-59 showed a histopathological proof of prostate cancer, and the figures went to 80% in men over 80, although only 1 in 26 (3.8%) would actually die of this disease.

In other words, it is more likely for men to die of this disease. This is especially important when considering a population screening within asymptomatic men.

## Disease progression and diagnosis [**3**]

The natural onset of prostate cancer is poorly understood, but the disease progression seems to be determined by the stage and the grade of the tumor.

Prostate cancer can be diagnosed by the following:

• Digital rectal examination (DRE)

• And/ or Trans-rectal ultrasound (TRUS)

• Serum PSA (Prostate-Specific Antigen): total PSA and free-PSA/ total PSA ratio

• With biopsy confirmation.

Each test can identify only a proportion of cancers, with higher rates of detection when used combined.

These tests were also used to determine the grade of the tumor and implicitly the prognosis and curability.

Unfortunately, establishing the clinical stage was insufficient, with approximately half of the tumors advancing after surgery.

## Therapies and screening [**3**]

There are three major therapy options in localized prostate cancer:

• Radical prostatectomy

• Radical radiotherapy and

• Conservative management (only monitoring and treating the symptoms).

Although the number of the radical approaches is continuously increasing, nowadays we do not have conclusive proofs/ studies regarding their comparative efficiency and cost versus benefit ratio.

Observational studies on small groups of patients suggest that mortality post-radical therapies are somewhat smaller when compared to the conservative management, although the mentioned studies did not provide data regarding post-treatment complications or quality of life in any of these approaches.

At present, there are intense debates concerning the opportunity of screening healthy men by testing the PSA levels.

The US PLCO (Prostate-Lung-Colorectal-Ovarian) Cancer Screening Trial [**4**] concluded that mortality was similar within the group in which screening for the PSA level was performed (followed by biopsy in cases considered at risk on that basis, then radical treatment in confirmed cases) versus the group without any screening (and only with conservative management), whilst the European trial ERSPC [**5**] argued that a decrease in the specific mortality was noticed in men screened for PSA levels versus the men who weren’t.

Observational studies suggested that digital rectal examination and PSA level, combined with trans-rectal ultrasound (and biopsy in suspicious cases) identified 3-5% “de novo” localized prostate cancers in men over 50 without any symptom, although it is worth mentioning there were both false positive and false negative results reported.

A series of key elements regarding the natural course of the disease, potential costs (financial, social and psychological) still remain to be assessed for a possible screening programme, and also the efficiency and cost-benefit ratio in treating the localized disease. Lack of consistent data (scientifically validated) and the major impact these problems may have, results in a general screening of prostate cancer in men not being recommended.

## Aetiology [**6**]

There is a broad spectrum of studies aiming to demonstrate causes and risk factors in prostate cancer development. 

Generally, the agents causing the onset of prostate cancer are unknown. A small number of risk factors have been identified, but they are supported by a large number of small studies, leading to contradictory or inconclusive results.

Most likely, prostate cancer is due to a complex interaction of:

• Age

• Endogenous hormone balance

• Genetic (predisposing) factors and

• Environment factors, including a fatty diet.

## Hormones [**7**]

Sexual hormones have been historically associated with prostate cancer, yet scientific evidence are inconsistent to decide if their involvement is aetiological or a phenotype component of the disease (cause versus effect).

Multiple studies assessed that:

• Steroid hormones’ receptors are overexpressed in prostate cancer cells

• Oestrogen therapy has proven effective in treating the prostate cancer

• Levels of testosterone and dihydrotestosterone are increased in the prostate cancer tissue versus the normal prostate tissue.

All these suggest the major importance of sex hormones in the pathogenesis of prostate cancer. Furthermore, the incidence of prostate cancer in castrated men is very low.

A series of studies assessed if sexual activity and prostate cancer were associated, but their results were inconclusive. Even their validity was questioned due to the inclusion of some confusion factors, as the fertility measured as the number of descendants, or sexual activity assimilated to the civil status (e.g. married).

Hormones’ involvement in prostate cancer pathogenesis makes it difficult to precisely determine the risk factors, due to the complex interactions among several hormones (testosterone, 5-alpha reductase, sex hormone binding globulin (SHBG), oestrogens), and environment factors (diet, smoking, etc.).

## Genetic factors/ family history [**8**,**9**]

Several studies established that genetic factors (as “hereditary”) are involved in the aetiology of some cases of prostate cancer.

A positive family history of prostate cancer has been identified as a significant risk factor in many studies. An odds ratio ≥ 2 for men with one first or second degree relative affected, has been calculated, the risk increasing with the total family members affected.

A family history of prostate cancer has been reported in 10% of the diagnosed men.

A family history of breast cancer also associates an increased risk of developing prostate cancer.

An epidemiology study revealed two types: hereditary prostate cancer, having an autosomal dominant trait and an early onset (Pack RS. Epidemiology of prostate cancer; with emphasis on familial clusters), and familial prostate cancer, with an obvious familial clustering but no noticeable mendelian trait.

These two types must be understoood as a pedigree analysis and not as a molecular substrate (where, as shown below, clues are a lot more complex).

Hereditary prostate cancer is supposed to lay its basis on an altered gene (with an autosomal dominant trait) which increases a lot the susceptibility of developing the disease (with incomplete penetrance). It includes a high percentage of the early onset cases and about 9% of the total cases of prostate cancer.

Familial prostate cancer is supposed to be a different form of the disease, more aggressive than in the general population (due to its tendency of an early onset), being responsible for a substantial proportion of the early onset cases.

The high risk given by a positive family history has emphasised the necesity of screening and early diagnosis programmes in men with a father or brother with prostate cancer.

## Environment factors [**9**]

The relationship between a disease and nutrition is often complex and difficult to interpret.

Still, several constituents of the diet seem to be associated with prostate cancer, especially the fat and the grain intake.

The clinical incidence of the prostate cancer is generally higher in the “western” populations. Studies demonstrated that mortality due to prostate cancer is highly associated with a high intake of fat, same as in the breast cancer, especially animal fat rather than vegetable fat. Overall, the total fat intake seems to be associated with a higher risk of developing prostate cancer, with a reported odds ratio of 1.6-1.9 and up to 3.6 in case of increased saturated fat intake. Similarly, when fat plasma levels were measured, both in the clinical and control groups, the odds ratio for men with high levels of alpha-linolenic acid was 3.0. The mechanism by which the fat intake is associated with a higher risk, is presumed to be the fact that the fat plasma excess can alter the normal synthesis of sex hormones, which would influence the development of prostate cancer.

The evidence concerning other nutritional factors are inconsistent, foods containing fibers and anti-oxidants as potential protecting factors (soy, vegetables, and fruits). Deficiency in A vitamin leads to a high risk of prostate cancer.

Several studies analyzed the exposure to a series of environment factors concerning the relative risk to develop prostate cancer. The only factor consistently associated is cadmium. Other possible agents have been found in the industry of rubber, exposure to radiations of different kinds, farm work, printing industry, and plumbing. These suggested studies have not been confirmed by other studies; on the contrary, a Scottish study on a group of men exposed to radium-226 indicated numbers significantly lower than the estimates.

## Histopathology [**10**]

There is a remarkable heterogeneity of the neoplastic regions, also multifocal, in prostate cancer, which leads to a number of serious problems, both diagnostically and etiologically.

Heterogeneity – the histopathological examination of cancer tissue usually shows an overlapping benign glandular tissue, prostatic intraepithelial neoplastic (PIN) tissue and a well-differentiated adenocarcinoma with various degrees of severity. Considering this aspect, in 1992 [**11**], Gleason proposed a system to establish the degree of the tumor, which is currently by far the most used by the pathologists, being an excellent predictor. In this system, a general score is obtained by adding the grades of the two most prevalent neoplastic lesions (e.g. 3+3; 3+4); a higher Gleason degree meaning a more advanced carcinoma.

Neoplastic lesions are multifocal – taken from a section of a biopsy, neoplastic regions are genetically different (non-clonal), even those next to each other (Bostwick 1998, Macintosh 1998). This observation suggests that multiple neoplastic regions can arise and evolve independently, making it difficult to establish the mechanisms of disease progression.

Practically, the heterogeneous and multifocal neoplastic areas, combined with the relatively small dimensions of the prostate, are a major impediment in obtaining enough homogenous material for molecular testing. These factors have been the major obstacles in identifying the genes and molecular processes involved in carcinogenesis and cancer progression. However, these difficulties have been overcome by the new techniques of microdissection and laser-capture microscopy of individual neoplastic lesions (Emmert-Buck 1996; Macintosh 1998), as well as cell-sorting techniques (Liu 1999), allowing an isolation of a relatively homogenous neoplastic population.

**Fig. 1:**
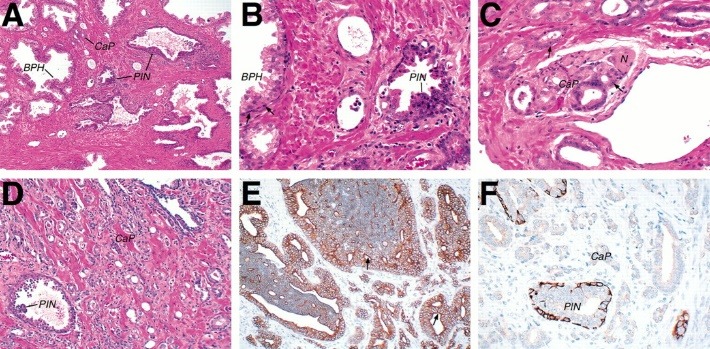
Histology of human prostate tissue

Panels A–D depict hematoxylin-eosin stains, while panels E and F show immunohistochemical analyses. (A) Low-power view showing the characteristic heterogeneity of prostate tissue, with this region containing a combination of BPH, PIN, and well-differentiated adenocarcinoma (CaP). (B) High-power view of a region in panel A, showing details of BPH and PIN. The region of BPH has ducts surrounded by basal cells (arrows), which are not found in the region of PIN. The area of PIN shows a transition within the same duct between normal and atypical hyperchromatic cells that contain larger nuclei with prominent nucleoli. (C) High-power view showing a nearby area of human prostate with a well-differentiated adenocarcinoma that is invading the peri-neural space (N marks the position of the nerve fiber). Note that the carcinoma cells have large nuclei with very prominent nucleoli (arrows). (D) View of a different prostate sample with a high-grade PIN and a mixture of Gleason grade 4 and 5 carcinoma in the rest of the field. (E) Immunohistochemical staining of PIN and carcinoma using anti-cytokeratin 8, which marks all of the epithelial cells. These PIN lesions have a cribriform pattern (arrows), but are still within the confines of a prostatic duct. (F) Immunohistochemical staining of a tissue section containing both PIN and carcinoma using anti-cytokeratin 14, which marks the basal cells. Notably, the PIN displays inconsistent staining, whereas the carcinoma has no staining. All the panels and interpretations were generously provided by Dr. Regina Gandour-Edwards and Dr. Robert Cardiff (School of Medicine, University of California–Davis, Davis, CA).

**Observation**

The origin cell of prostate cancer is currently not elucidated.

The prostate epithelium consists of basal, intermediate, luminal and neuroendocrine cells.

The controversy regarding the origin cell of prostate cancer is not new. Anterior studies indicated that this disease has two types of origin cells: the luminal secretory cells, or the basal cells, respectively.

The neoplastic tissue consists only of luminal, and not basal, cells, so that the majority of pathologists supposed that the disease is of luminal origin.

However, in normal prostatic tissue, the cells have a great regenerative potential, in contrast with the luminal cells, so that the majority of biologists were in favor of the basal origin.

For a long time, there has been no explanation regarding the way a basal cell can transform into a luminal cancer cell.

In July 2010, Owen Witte, MD [**12**], from the University of California, Los Angeles, published an experiment in Science magazine, in which human basal prostatic cells transformed into cancer cells, and then transplanted in mice, were able to generate luminal cells.

However, luminal tumors can originate from normal luminal cells, at least in mice. Michael Shen published an equally convincing study in Nature magazine [**13**] detecting the origin of prostate cancer in mice (not using human cells), as from a rare population of luminal cells (castration-resistant Nkx3.1-expressing cells CARNS) which, he has demonstrated, can regenerate the normal prostatic tissue, giving birth both to basal and luminal cells. After the deletion of the PTEN gene (tumour suppresor) in castrated mice in which androgens were administered, these CARNS rapidly evolved in tumours with a high rate of growth. CARNS can be the origin cells of prostate cancer.

**Observation **

These hypotheses do not exclude each other and neither other possibilities.

**Chromosome abnormalities in neoplastic prostate tissue**

Human prostate cancer is characterized by multiple alterations as chromosomes’ structure (huge mutations), involving a large number of chromosomal regions, although the genes on which these processes are based are unknown.

Still, prostate cancer remains poorly characterized cytogenetically. Partly due to method problems (they are difficult, choosey, tumor cultures), and partly due to the decreasing number of radical prostatectomies, so far they are the main source of material for a cytogenetic analysis.

Pan Y & co. [**14**] (Urology Department, Karolinska Hospital, Stockholm, 1999) have studied the chromosomal makeup of three well-known neoplastic cell lines in prostate cancer: LNCaP, PC-3 and DU145, using spectral karyotyping (SKY). The SKY analysis revealed complex configurations for each of the three lines, with 87, 58/ 113, and 62 chromosomes, respectively. All cell lines carried alterations of chromosomes 1, 2, 4, 6, 10, 15 and 16; still, they did not detect recurrent breakpoints. In comparison with anterior studies which used CGH (Comparative Genomic Hybridization), SKY analysis pinpointed the existence of several balanced translocations and their rearrangement breakpoints. SKY analysis has been validated by using FISH (Fluorescence In Situ Hybridization), with both chromosome-specific and locus-specific probes.

Identifying chromosomal alterations in the neoplastic cell lines can become useful in the attempt of cloning the genes involved in the oncogenesis of prostate cancer.

The gene mutations in the prostate neoplastic tissue – association studies conducted so far [**15**] are the following:

EEF1A1L14 (PTI1 ) - EEF1A1L14 and Prostate Cancer

HPC1 (PCS1) 1q24-q25 - HPC1 and Familial Prostate Cancer

PRCA1 1q24-q25 - PRCA1 and Prostate Cancer?

PCAP (HPC2) 1q42.2-1q43 - PCAP and Familial Prostate Cancer

SRD5A2 2p23 - SRD5A2 and Prostate Cancer

BIN1 (AMPHL , SH3P9) 2q14 - BIN1 and Prostate Cancer

CTNNB1 (CTNNB) 3p22-p21.3 - CTNNB1 and Prostate Cancer

TGM4 (pTGase) 3p22-p21.33 - TGM4 Expression and Prostate Cancer

ACPP 3q21-q23 - PAP (ACPP) Expression and Prostate Cancer Prognosis

FGF2 (FGFB , BFGF) 4q25-q27 - FGF2 Over-Expression in Prostate Cancer

FGF1 (FGFA) 5q31 - FGF1 Over-Expression in Prostate Cancer

SPARC (ON , OSN , BM-40) 5q31.3-q32 - SPARC Expression and Prostate Cancer

VEGF (VEGFA) 6p12 - VEGF and Prostate Cancer

CDKN1A (P21 , WAF1 , CIP1) 6p21.2 - CDKN1A Expression in Prostate Cancer

IL6 (IFNB2) 7p21 - IL6 and Prostate Cancer

CYP3A4 7q22.1 - CYP3A4 and Prostate Cancer

CAV1 (Caveolin-1) 7q31.1 - CAV1 and Prostate Cancer

PDGFRL (PRLTS) 8p22-p21.3 (?) - PDGFRL and Prostate Cancer

PSCA 8q24.2 - PSCA and Prostate Cancer

MSMB (PSP94 , PSP57) 10q11.2 - MSMB Expression and Prostate Cancer

PTEN (MMAC1 , MHAM , BZS) 10q23.3 - PTEN and Prostate Cancer

FGF8 (AIGF) 10q24 - FGF8 Over-Expression in Prostate Cancer

CYP17 10q24.3 - CYP17 and Prostate Cancer

MXI1 10q25 - MXI1 and Prostate Cancer

MKI67 (Ki-67) 10q25-qter - Ki-67 Expression in Prostate Cancer

KAI1 (CD82 , R2 , ST6) 11p11.2 - KAI1 and Prostate Cancer

POV1 (PB39) 11p11.2-11p11.1 - POV1 and Prostate Cancer

CDKN1B (KIP1 , P27) 12p13 - CDKN1B and Prostate Cancer

IGF1 12q22-q24.1 - IGF1 Expression in Prostate Cancer

TP53 (p53, P53) 17p13.1 - TP53 and Prostate Cancer

ERBB2 (HER2 , NEU) 17q11.2-q12 - ERBB2 and Prostate Cancer

PI5 (maspin) 18q21.3 - Maspin and Prostate Cancer

KLK3 (PSA , APS) 19q13 - Prostate Specific Antigen and Prostate Cancer

-Use of Percent Free PSA in Prostate Cancer Staging

KLK2 (hK2) 19q13 - KLK2 Expression and Prostate Cancer Prognosis

BGP1 (CD66A, C-CAM1, CEACAM1) 19q13.2 - CD66a and Prostate Cancer

ETS2 21q22.3 - ETS2 and Prostate Cancer

TMPRSS2 (PRSS10) 21q22.3 - TMPRSS2 and Prostate Cancer

AR (DHTR) Xq11.2-q12 - AR and Prostate Cancer

HPCX Xq27-q28 - HPCX and Hereditary Prostate Cancer

SRY Yp11.3 - SRY Deletion in Prostate Cancer

**Transcription factors (TF) and prostate cancer**

So far, we have known very little about the molecular mechanisms of controlling the gene expression, tissue-specific, and the way gene expression modifies the generating pathological states, including cancers. This aspect is largely due to the lack of information on “the second genetic code” – the code of gene expression, starting with the specificity of binding the transcription factors (TF) for some DNA sequences (in vivo they manifest a great variability).

TF act as activators or repressors of transcription, binding specific DNA sequences from the promoter or enhancer regions. At present, there are many known TF and their “preferences” to particular types of DNA sequences. Furthermore, it seems there are “motives of sequences” which correspond to these “preferences”, detected by a computer based genomic analysis.

We will discuss below one of the largest families of TFs, called ETS (E26), whose members have broad-spectrum functions, both physiological and oncogenic (Bartel 2000; Sharrocks 2001; Kumar-Sinha 2008). The first factor identified is ETS1, discovered as a homologue of the oncogene of the avian leukemia virus E26 in 1983 (Leprince 1983; Nunn 1983). Until now, a number of 27 members of the ETS family have been identified in men, respectively 26 in mice (Bult 2008).

The ETS factors play a role both in the development (Schober 2005) and in the functioning of tissues and cells (Bartel 2000). They are essential in angiogenesis, haematopoesis and neuron development (Bartel 2000; Vrieseling și Arber 2006). The cells’ reaction to the action of activated ETS factors include cell multiplying, differentiation and migration (Sharrocks 2001; Schober 2005), depending on the type and state of the target cell.

Translocations which alter the activity of some members of the ETS family are associated with many types of cancer in men. In observed cases of cancers with translocations, the ligation domain is lost, while the regulatory domain of the ETS is found in another class of TF, having another ligation domain, corresponding to another specific DNA sequence (e.g. ETV6-RUNX1; Golub 1995; Mavrothalassitis și Ghysdael 2000). Translocations leading to the fusion of a strong activating domain of transcription (of another TF) with the ligation domain of an ETS (e.g. EWS fused with FLI1 or ERG in Ewing sarcome - Delattre 1992; Sorensen 1994) and/ or an overexpression of a member of the ETS family, due to the insertion of a strong cis-activating element upstream of its gene (Tomlins 2005, 2007), are more common in cancers.

In fact, the most common translocation known to cancers is TMPRSS2-ERG fusion, which introduces a strong regulating element from the androgen receptor gene upstream of the ERG gene (Tomlins 2005). Together with the other translocations involving ETV1 and ETV4 of the same family, over 50% of the total prostate cancers show a hyperactivity of the ETS proteins (Kumar-Sinha 2008).

**SNPs used in studying the polymorphisms associated to the risk of prostate cancer**

A single-nucleotide polymorphism (SNP) is a variation of a DNA sequence in which a single nucleotide – A, T, C or G – within the whole genome (or within a common sequence) is different between the members of the same species or between the homologous chromosomes of the same individual. For instance, two DNA fragments originating from two different individuals have AAGCCTA and AAGCTTA sequences, meaning a difference of a single nucleotide (C or T). In this case, we state the fact that there are two alleles.

Genome-wide association studies, GWAS, identified more than 30 variants contributing to the susceptibility of developing prostate cancer [**16**-**19**], most discoveries being made in the population of European origin. However, as it was observed for most of the common diseases, the variants detected by GWAS characterize a small risk, either individually or combined, that have a limited value in predicting the disease. Most variants at risk for prostate cancer are localized outside the known genes, some in non-coding regions, and others in regions hosting more independent molecular signals (regions with overlapping genes). Therefore, for most of the risk loci, the genetic identity, frequency, and associated risk with biological relevant alleles are unknown. The variants at risk identified by GWAS have shown variability in frequency between races, populations, and ethnic groups. Even by lacking functional data, the variants associated with cancer risk can pinpoint the genetic basis of risk differences between populations (see the table with the ethnic analysis of the risk); a good example would be the variation of sequence at the 8q24 locus, which in the previous studies proved to be significant in justifying interethnic risk differences. Testing the risk variants and mapping at high resolution will probably help identify and localize several subsets of markers that better characterize the risk of functional alleles from loci at risk, also determining their contribution to differences between races, populations, ethnic groups more precisely.

Three studies published in “Nature Genetics” on February 10 2008 reported a double number of variants (SNPs) associated with the risk of prostate cancer, as 10 new variants were identified. Three independent groups of researchers have conducted the studies.

a. The first of them was conducted by Rosaline Eeles, MA, PhD, FRCP, FRCR [**16**] at the Cancer Genetics Unit, Royal Marsden NHS Foundation Trust, Surrey, UK. The purpose of the study was to develop a genetic profile at risk for prostate cancer. They succeeded in identifying a subgroup of 10% of the total patients (with prostate cancer, and control patients, respectively) as a combination of high-risk variants, meaning 2x the base risk of developing prostate cancer. Their significance in developing several more aggressive forms of prostate cancer is under evaluation.

Dr. Eeles & Co evaluated 1854 blood samples from British men with prostate cancer diagnosed at ages younger than 60 years old or having a family history of prostate cancer. The control group included 1894 men aged over 50 with a low PSA serum concentration (< 0.5 ng/ mL). They analyzed a number of 541129 SNPs, estimated to cover approximately 90% of the SNPs from HapMap (a catalogue of human genetic variants). To confirm the results, the research was replicated on other 3268 men with prostate cancer and 3366 control cases.

They identified 7 loci, significantly associated with prostate cancer on chromosomes 3, 6, 7, 10, 11, 19 and X, and confirmed the previous reports of association of loci 8q24 and 17q. Furthermore, they observed that 3 of the newly identified loci corresponded to candidate genes concerning susceptibility: MSMB (proximally), LMTK2 and KLK3.

**Observation**

MSMB encodes a marker, which would be interesting to evaluate versus PSA.

b. Within the second study (PLCO, Prostate-Lung-Colorectal-Ovarian Cancer Screening Trial in the US) [**4**], Stephen Chanock, MD, & Co identified new loci at risk on chromosomes 7, 10 (2 loci) and 11, and confirmed 3 previously reported loci (two independent SNP at 8q24 and in HNF1B). They included 1172 men with prostate cancer (484 non-aggressive and 688 aggressive) and 1157 control cases (with low PSA levels).

The study has been replicated by 4 other independent studies, having 26958 SNPs genotyped in 4020 (total) patients with prostate cancer versus 4028 controls. From the data analysis combined, resulted newly identified loci on chromosomes 7, 10 and 11 with a high significance and other 9, which were just suggestive for prostate cancer.

They also noticed that several loci on chromosome 10 correspond to MSMB gene, which encodes for a beta-micro-seminoprotein, considered a potentially new biomarker in prostate cancer.

c. The third clinical trial [**19**], a Genome-Wide-SNP-Association-Study (GWAS) for prostate cancer, included 23,000 men and took place in Iceland, being conducted by Kári Stefánsson, MD, Dr. Med., CEO and cofounder of deCODE Genetics. It has been followed by a replicating study on more than 15,500 men in Europe and USA.

The study identified 8 loci with a high significance of risk of prostate cancer.

Dr. Stefánsson & Co identified rs5945572 on Xp11.22 and rs721048 on 2p15, which are 2 new variants associated with prostate cancer. Also, the variants 2p15 showed a significantly stronger association than all the other with the aggressive forms of prostate cancer.

In 2008, DeCODE Genetics launched a lab test for the most common SNP variants associated with prostate cancer (and confirmed in several populations). The test consisted in detecting 6 og the SNP variants previously discovered plus the 2 newly identified.

**Observation**

Anyway, there are more genetic components in prostate cancer than in most other cancers.

**Prostate cancer – molecular and genetic aspects**

The Prostate Specific Antigen (PSA; also called kallikrein III, seminin, seminogelase, ɣ-seminoprotein and antigen P-30) is a 34 kD glycoprotein produced almost exclusively in the prostate.

Functionally, it is a serine-protease (EC 3.4.21.77), and the gene encoding it is localized on chromosome 19q13 in humans.

PSA secretion by the human neoplastic cells LNCaP is influenced by acute stimuli like the Vasoactive Intestinal Peptide (VIP), GHRH (growth hormone-releasing hormone) and chronic stimuli like the androgens [**20**].

The gene expression of PSA androgen-induced in LNCaP cells is mediated by dihydrotestosterone (DHT) in a dose-dependant manner [**21**].

**Androgen Receptor Gene [**22**,**23**]**

The androgen receptor gene (AR – located on the X chromosome) has been studied by means of the genetic variants both in relation with the risk of developing prostate cancer and the progression of the disease. The AR is expressed during all the stages of carcinogenesis. A study demonstrated that men with hereditary prostate cancer who followed radical prostatectomy had a higher percentage of cells expressing AR and lower percentage of cells expressing the alpha-receptor for oestrogen, compared to men with sporadic prostate cancer. The authors suggested there might be a specific pattern of expression of the AR associated with hereditary prostate cancer.

**5-alpha-reductase (SRD5A2) [**24**]**

Epidemiological molecular studies examined correlations between the polymorphisms of 5-alpha-reductase gene of type II, which is also involved in the metabolic cycle of the androgens. There are 2 isoenzymes of the 5-alpha-reductase. The gene encoding 5-alpha-reductase type II (SRD5A2) is located on chromosome 2. It expresses in the prostate, where testosterone is irreversibly converted to dihydrotestosterone (DHT) by this enzyme. There are clues that the 5-alpha-reductase type II has a low activity in populations with a small risk of prostate cancer, as Asian men (Chinese and Japanese).

**Oestrogen Beta-Receptor gene [**25**]**

In a population study performed in Sweden, of 1415 cases of prostate cancer and 805 control cases, of corresponding ages, the association of several SNPs from the oestrogen beta-receptor gene (ER-beta) with prostate cancer has been analyzed. A SNP located in the promoter region of the ER-beta, rs2987983, has been associated with a global risk of prostate cancer of 1.23, and localized prostate cancer of 1.35. This study has not been replicated.

**E-Cadherin gene [**26**]**

E-Cadherin is a tumor suppressor, which was better studied in some forms of gastric carcinoma. A certain SNP called -160→A, located in the promoter region of the E-cadherin gene, that alters the transcription of the gene, is currently evaluated within a meta-nanalysis of 26 case-control studies for being involved in the aetiology of 7 types of invasive cancers.

**Toll-like receptor genes [**27**-**29**]**

The family of the Toll-like receptors (TLR) has been recognized as a critical component of the innate immune system (the Nobel Prize in Medicine and Physiology, 2011) and they have been thoroughly analyzed by means of their modifications in autoimmune diseases. Lately, research has been focusing more on their involvement in carcinogenesis. Results have been contradictory, varying from a low risk, to null association, or high risk.

**Recent advances in Prostate Cancer therapy**

**Biomarkers were identified to have an impact on disease progression**

As generally known from all previous observations and studies referring to prostate cancer, this disease evolves particularly slow and can have a minor impact on the patients’ quality of life or mortality rate. Just a relatively small proportion of all the diagnosed prostate cancers (about 10% according to the researchers at Harvard University), extend aggressively beyond the prostate capsule and determine life-threatening situations. However, how to predict, by any biological measurement, which will be indolent and which aggressive, has always been a challenge for professionals.

Recently, researchers from the University of Copenhagen, in cooperation with several institutions in Sweden [**30**], having analyzed over 9000 prostate protein levels, found and validated a biomarker which can successfully be used in the prognosis of disease progression. This biomarker, a tissue-based neuropeptide called Pro-NPY (pro-neuropeptide), shows increased levels in high-risk cases and low levels in indolent cancers. It also shows specificity for prostate cancer. This biomarker can draw a line between aggressive cases, that require immediate intervention, and cases that are slow and will benefit from a simple observation.

## References

[1] NCI Surveillance Epidemiology and End Results (SEER).

[2] Prostate cancer - UK and worldwide incidence statistics. http://info.cancerresearchuk.org/cancerstats/types/prostate/incidence/#geog.

[3] (2015). Prostate cancer. Fort Washington, Pa.: National Comprehensive Cancer Network. http://www.nccn.org/professionals/physician_gls/f_guidelines.asp.

[4] (2012). J Natl Cancer Inst.

[5] (2009). N Engl J Med.

[6] Potential causes of prostate cancer. http://www.macmillan.org.uk/information-and-support/prostate-cancer/early-prostate-cancer/diagnosing/causes-and-risk-factors/potential-causes-of-cancer/prostate-cancer-causes.html.

[7] (2007). Urol Int.

[8] Pack RS (1993). Epidemiology of prostate cancer; with emphasis on familial clusters. Cancer Bull.

[9] American Cancer Society.

[10] Abate-Shen  C, Shen  MM (2000). Molecular genetics of prostate cancer. Genes Dev.

[11] Gleason  DF (1992). Histologic grading of prostate cancer: a perspective. Hum Pathol.

[12] (2010). Science.

[13] (2009). Nature.

[14] (1999). Cytogenet Cell Genet.

[15] Genetics Home Reference.

[16] Eeles  RA, Kote-Jarai  Z, Giles  GG, Olama  AA, Guy  M, Jugurnauth  SK, Mulholland  S, Leongamornlert  DA, Edwards  SM, Morrison  J, Field  HI, Southey  MC, Severi  G, Donovan  JL, Hamdy  FC, Dearnaley  DP, Muir  KR, Smith  C, Bagnato  M, Ardern-Jones  AT, Hall  AL, O’Brien  LT, Gehr-Swain  BN, Wilkinson  RA, Cox  A, Lewis  S, Brown  PM, Jhavar  SG, Tymrakiewicz  M, Lophatananon  A, Bryant  SL (2008). Multiple newly identified loci associated with prostate cancer susceptibility. Horwich A, Huddart RA, Khoo VS, Parker CC, Woodhouse CJ, Thompson A, Christmas T, Ogden C, Fisher C, Jamieson C, Cooper CS, English DR, Hopper JL, Neal DE, Easton DF. UK Genetic Prostate Cancer Study Collaborators; British Association of Urological Surgeons’ Section of Oncology; UK ProtecT Study Collaborators. Nat Genet.

[17] Zheng  L, Sun  J, Wiklund F, Smith S, Stattin  P, Li  G, Adami  HO, Hsu  FC, Zhu Y, Bälter K, Karim Kader  A, Turner  AR, Liu  W, Bleecker  ER, Meyers DA, Duggan D, Carpten  JD, Chang  BL, Isaacs WB, Xu  J, Grönberg  H (2008). Cumulative Association of Five Genetic Variants with Prostate Cancers. N Engl J Med.

[18] Haiman  CA, Chen  GK, Blot  WJ, Strom  SS, Berndt  SI (2011). Characterizing Genetic Risk at Known Prostate Cancer Susceptibility Loci in African Americans. PLoS Genet.

[19] (2008). Nat Genet.

[20] Zoltan  Rekasi (2001). Tulane University School of Medicine, The Prostate.

[21] Zhu  YS (2003). Department of Medicine/ Endocrinology, Weill Medical College of Cornell University. Journal of Andrology, New York.

[22] Cunningham  JM, Hebbring  SJ, McDonnell  SK (2007). Evaluation of genetic variations in the androgen and estrogen metabolic pathways as risk factors for sporadic and familial prostate cancer. Cancer Epidemiol Biomarkers Prev.

[23] Lindström  S, Zheng  SL, Wiklund  F (2006). Systematic replication study of reported genetic associations in prostate cancer: Strong support for genetic variation in the androgen pathway. Prostate.

[24] (2014). Mol Med Rep.

[25] Thellenberg-Karlsson C, Lindström  S, Malmer  B, Wiklund  F, Augustsson-Bälter  K, Adami  HO, Stattin  P, Nilsson  M, Dahlman-Wright  K, Gustafsson  JA, Grönberg  H (2006 ). Estrogen receptor beta polymorphism is associated with prostate cancer risk. Clin Cancer Res.

[26] Wang  GY, Lu  CQ, Zhang RM, Hu XH, Luo ZW (2007). The E-cadherin Gene Polymorphism 160C/A and Cancer Risk: A HuGE Review and Meta-Analysis of 26 Case-Control Studies. American Journal of Epidemiology.

[27] Sun  J, Wiklund  F, Zheng  SL (2005). Sequence variants in Toll-like receptor gene cluster (TLR6-TLR1-TLR10) and prostate cancer risk. J Natl Cancer Inst.

[28] Chen YC, Giovannucci E, Kraft  P (2007). Association between Toll-like receptor gene cluster (TLR6, TLR1, and TLR10) and prostate cancer. Cancer Epidemiol Biomarkers Prev.

[29] Stevens  VL, Hsing  AW, Talbot  JT (2008). Genetic variation in the toll-like receptor gene cluster (TLR10-TLR1-TLR6) and prostate cancer risk. Int J Cancer.

[30] Iglesias-Gato  D, Wikström  P, Tyanova  S, Lavallee  C, Thysell  E, Carlsson  J, Hägglöf  C, Cox  J, Andrén  O, Stattin  P, Egevad  L, Widmark  A, Bjartell  A, Collins  CC, Bergh  A, Geiger  T, Mann  M, Flores-Morales  A The Proteome of Primary Prostate Cancer. European Urology.

